# Social Robots for Meaningful Seated Activities: Acceptance & Use by Older Adults

**DOI:** 10.3390/healthcare12131334

**Published:** 2024-07-04

**Authors:** James R. Sadler, Aila Khan, Michael Lwin, Omar Mubin

**Affiliations:** 1 School of Business, Parramatta City Campus, Western Sydney University, Parramatta, NSW 2150, Australia; m.lwin@westernsydney.edu.au; 2 School of Computer, Data and Mathematical Sciences, Parramatta City Campus, Western Sydney University, Parramatta, NSW 2150, Australia; o.mubin@westernsydney.edu.au

**Keywords:** healthy aging, older adults, social robots, meaningful activities, quality of life

## Abstract

Healthy aging requires the maintenance of good physical and cognitive activity. However, as they age, older adults often experience a decline in physical and cognitive activity, leading to a more sedentary lifestyle. Some older adults may not have a choice but to become increasingly sedentary as they age due to injury or deteriorated physicality. As such, they require assistive technologies to aid in their daily lives and activities to maintain healthy cognitive function. Social Robots are a newer form of assistive technology, specifically designed for social interactions and gameplay. As with other assistive technologies, compliance barriers to their acceptance and use for meaningful, seated activities among older adults are expected. To better explore this phenomenon, improve quality of life and understand what drives older adults to accept and use newer forms of technology like social robots, this conceptual paper conjoins two theoretical frameworks: The Activity Theory of Aging (ATA) and the Unified Theory of Acceptance and Use of Technology (UTAUT). As social robots hold great promise for improving the quality of life for older adults, exploring what driving factors could enable their greater acceptance and use is essential to furthering this field of study within Australia.

## 1. Introduction

Maintaining quality of life for older adults is a vital component of healthy aging [1]. Maintaining good physical and cognitive activity is essential to ensure quality of life and healthy aging [2]. However, it is well established that as older adults age, they ordinarily disengage from social interactions, become increasingly sedentary and decline both physically and cognitively, especially in their later years [3]. The combination of declines in real-life social interactions and increasingly sedentary lifestyles are antithetical to healthy aging among older adults, increasing the prevalence of a range of physical and cognitive ailments that decrease the quality and quantity of their lives [2,4].

While a sedentary lifestyle is generally frowned upon by public health experts as antithetical to healthy aging [5,6], some older adults might not have a choice. This can be due to injury, loss of mobility or any other ailment that may cause an older adult to remain in a sedentary activity state [7]. Sedentary behaviours generally refer to activities that do not increase energy expenditure substantially above the resting level, for example sitting, lying down, or watching television [8]. However, if older adults are engaged in seated activities associated with an increased ‘executive function’ these may result in greater positive health outcomes [6,8]. ‘Executive functions’ (EFs) are an umbrella term related to higher-order cognitive processes that require focus, attention, goal orientation and the inhibition of impulses or interference [9]. Types of seated activities can transcend passive behaviours and utilise EFs [6]. These can include listening to music, solving a puzzle, playing a game, or reading [6,10,11]. These forms of seated activities that engage EFs can create positive cognitive and psychological outcomes for older adults who may not have a choice to have a sedentary lifestyle [5,6].

To employ meaningful seated activities and newer forms of assistive technologies among older adults, the focus of many exploratory studies is on their acceptance and use [10,11,12]. Subsequently, this publication will seek to outline a theoretical basis for proposing what will drive or motivate older adults to accept and use newer forms of assistive technology for meaningful seated activities.

## 2. Social Robots as Assistive Technologies

Assistive technologies are aids or devices designed to help older adults who have physical or cognitive deficits [12]. Social robots are a newer form of assistive technology, specifically designed to aid in social interactions or toward certain goals like gameplay or motivation to engage [13]. Social robots are machines, equipped with a degree of artificial intelligence and human-like mechanics that can mimic or replicate a human interaction with a user [14]. These interactions include touch, handshaking, greetings, verbal interactions, gameplay and recognising cues. Social robots take a variety of forms, including quasi-animals and humanoids [14].

Social robot use among older adults is increasingly ubiquitous. The designation/setting for a social robot deployment among older adults ultimately depends on its design [14]. As social robots’ primary function is for social interactions, their use among older adults has a pronounced focus on companionship [15]. This was found by Sun & Liu [16], whose research indicated an improvement in the quality of life for older adults by using social robots to provide companionship. As demonstrated by Khosla et al. [17], interactions between social robots and older people found significant improvements in observed states of well-being. This is reinforced by Ge & Schleimer [18], whose analysis of older adults’ use and acceptance of social robots demonstrated a marked improvement in general well-being.

**Figure 1:**
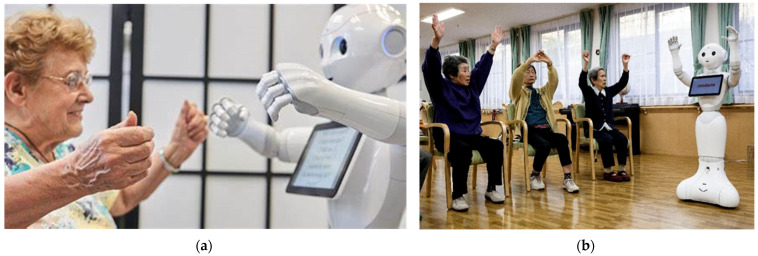
(**a**,**b**) Social Robot ‘PEPPER’ Engaging Older Adults in Meaningful Seated Activities (Credit: Getty Images).

Other benefits and design features of social robots include their ability to engage or motivate older adults toward certain tasks or goals. These are usually called socially assistive robots (SARs) and can be deployed among older adults to aid in motivation behaviours to engage [19]. For example, engage an older adult in an exercise routine, toward a social interaction with others or engage them in gameplay or a seated activity (Figure 1a). This is consistent with Feil-Seifer & Mataric [19] and Kyong et al. [20], who demonstrated that SAR deployment among older adults can positively influence motivation to engage in an exercise routine. Furthermore, Macis et al. [21], demonstrated that the deployment of a SAR among older adults can improve the motivation to engage and maintain a daily stretching and tai chi routine. Pharmacological disbursement is another area where SARs have been deployed with effectiveness. According to Smarr et al. [22], reminders to take medications and management systems for medications by SARs have produced positive outcomes and a greater level of compliance among older adults.

### Acceptance of Social Robots by Older Adults

Assistive technology use among older adults is commonplace as it can bring some normalcy back into their lives following an injury or negative health diagnosis [23]. While the benefits of using assistive technologies are clear, their regular use by older adults can be challenging [24]. Resistance, hesitancy, and non-compliance with the use of interventions like assistive technology are commonplace among older adults [25]. Depending on the type of assistive technology this resistance by an older adult can be due to many factors. This includes a perceived loss of autonomy, fear of change, history or experience, loss of motivation or drive, decline in cognition, religious or belief reasons, and/or any other subjective perception that an older adult may have to not want to engage or use the assistive technology [26,27]. As social robots are a newer form of assistive technology [14]., their benefits to older adults are still being explored. Subsequently, barriers to their acceptance and use by older adults have been documented and are expected in future research [28].

## 3. Research Scope

### Publication Aims

The use of social robots among older adults is an emerging field of study in an Australian context. As with many other interventions and assistive technology, hesitancy, or resistance to their use by older adults are expected. Subsequently, the exploratory research being conducted in Australia on social robots and older adults is primarily concerned with understanding what will drive their acceptance and use.

The main aim of this paper is to provide a combined conceptual framework, using the well-established ATA and the UTAUT models that may provide insight into the factors influencing older adults’ acceptance of using social robots for meaningful seated activities.

## 4. Theoretical Frameworks

### 4.1. The Activity Theory of Aging (ATA)

Concerns over the quality of life for older adults and what constitutes healthy aging were first documented within the ATA. This theory proposes that well-being and longevity for older adults depend on the quality of their social interactions and maintaining an active lifestyle [2]. This is particularly important in an individual’s later years [29]. In contrast, a deterioration of social relationships and a decline in activity have demonstrated a decrease in the quality and quantity of life for an older adult [2].

The older population is set to increase exponentially and move into assisted living facilities. This has raised concerns for clinicians over how to maintain a healthy aging protocol through activity and social connections in aged care facilities. As highlighted by Peri et al. [1], mobility into a care facility can reduce the autonomy of an older adult, further declining their social interactions and daily activities [4]. Using the ATA as a theoretical basis and foundational to this research, this article proposes the use of social robots by older adults for engagement in meaningful seated activities and social interactions.

### 4.2. The Unified Theory of Acceptance & Use of Technology (UTAUT)

Research on social robots and older adults is in its early stages of study in Australia. While they hold promises to improve the quality of life for older adults, acceptance of robots and their use by this demographic poses an obstacle [29]. Generally, older adults have lower adoption rates of newer technologies [30], have a difficult time navigating the technology and are more likely to disengage [30]. Among the 3.7 million older adults in Australia, over 38% had not used the internet or digital technology within the last 3 to 6 months [31]. This is consistent with other forms of technology used among older adults. This is common with assistive technology used by older adults for clinical aged care [25].

**Figure 2:**
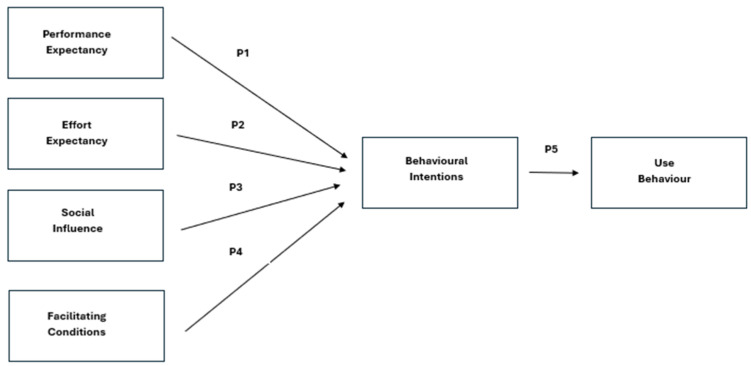
Model Adopted from the Unified Theory of Acceptance & Use of Technology.

The Unified Theory of Acceptance and Use of Technology (UTAUT) is the conceptual framework at the basis of this research (Figure 2). Namely, what driving factors will contribute to an older adult’s acceptance and use of assistive technologies like social robots? Other studies that have used the UTAUT model to assess the acceptance and use of assistive technologies by older adults indicate similar results. Consistent with Hsu et al. [32], older adults are usually the last in a population to adopt newer technologies, even if they can bring a tangible benefit to their quality and quantity of life. This was explored by Bixter et al. [30], who investigated what would drive older adults to be more likely to adopt social media technologies using the UTAUT model. Subsequently, the UTAUT model provides a robust framework for understanding the motivating factors that will drive older adults to accept and use newer forms of assistive technologies like social robots. The UTAUT model used in this context has been modified to include four primary concepts that researchers believe are imperative in driving behavioural intentions: Performance Expectancy, Effort Expectancy, Social Influence and Facilitating Conditions [32].

## 5. Theoretical Propositions

### 5.1. Performance Expectancy

Performance Expectancy relates to whether an individual believes that a system or technology will help or benefit them [32]. This concept is broad, can be expressed in multiple ways and applies to a range of circumstances regarding technology acceptance and use by an individual [32]. Performance Expectancy is also foundational in other models such as the Technology Acceptance Model (TAM). Conversely, in the TAM model, it is referred to as “Perceived Usefulness” [33]. As defined in TAM, this concept relates to the degree an individual believes using the technology or system will help them [33]. Simply, if an individual believes that the technology is useful for him or her, then the likelihood of using the technology is high [34].

Older adults are sometimes unaware of how a specific technology can benefit them or what types of technologies are available for them to use [35]. This is explained by the Life Course Theory. The Life Course Theory, as proposed by Glen Elder provides insight into older adults’ technology perceptions and their usage over some time considering previous event histories that may influence later outcomes in individuals’ lives [36]. Older adults did not grow up with these types of technologies that may be prevalent in their lives today [37]. Therefore, their interaction with these technologies then was non-existent and their expectations from these forms of technology may also be limited [35].

On the other hand, if older adults are exposed to technology such as social robots and are provided with an opportunity to familiarize themselves with the use of this technology, for example undertaking seated activities or socially interacting, then researchers propose that this would increase older adults’ intentions to use this technology. Subsequently, this will also drive their self-efficacy in the technology and would be more likely to continue its use and recommend it to their peers [38].

**Proposition** **1.** *Older adults’ expectations regarding a social robot’s usefulness will affect their intentions to use the humanoid.*

### 5.2. Effort Expectancy

Effort expectancy or ‘perceived ease of use’ relates to whether an individual perceives the technology as easy to adopt or use, for themselves and/or others [32]. Among Australia’s older adults, effort expectancy ordinarily raises many challenges due to older adults’ general averseness to technology [30]. For example, whereas older adults may be able to comprehend the benefits of assistive technology, they may perceive the product, service, or device as being difficult to use and not want to try it [39].

As people age, they begin to experience problems with their overall health, including cognitive functions [40]. The cognitive load theory first proposed by John Sweller helps to explain the reason. This theory is based on the amount of information a human can hold and analyse [41]. Highlighted by Van Gerven et al. [42] there are three types of cognitive declines that seniors experience: (a) a decrease in working memory capacity, which means short-term memory loss; (b) a decline in the rate at which information is processed and understood, which means it takes longer for older adults to comprehend information; (c) a decline in the ability to ignore irrelevant information, which means older adults may get distracted with information which is not relevant to a task.

In the context of this research, ‘effort expectancy’ refers to older adults’ perceptions regarding the amount of effort required to use a social robot. The greater the amount of effort needed, the less likely they are to want to engage in it. On the other hand, if older adults are exposed to technology such as social robots and are provided with assistance and time to use and build experience with this technology, then researchers expect that this would assist older adults in using the robot, and consequently increase their intentions to use a social robot. As older adults’ belief in their capability to use a robot increases, they would be more likely to continue its use and recommend it to their peers [38]

**Proposition** **2.** *Older adults’ perceptions regarding the ease with which a social robot can be used will be linked to their intentions to use the humanoid social robot (for meaningful seated activities).*

### 5.3. Social Influence

Social influence, also referred to as ‘social norm’ is defined as the degree to which an individual perceives that ‘important others’ believe he or she should use the new technology [32]. ‘Important others’ can include an individual’s peers, partners, colleagues, and media consumed [43], and even superiors in mandatory settings [32].

The Disengagement Theory [3] (p. 227) states that “aging is an inevitable, mutual withdrawal or disengagement, resulting in decreased interaction between the aging person and others in the social system he belongs to”. The theory claims that it is natural and acceptable for older adults to withdraw from society [44]. However, it should be noted that the older adult cohort could still be influenced by people beyond family and close friends. In an aged care setting, older adults usually interact with carers, healthcare professionals, volunteers, and other residents of the care facility [45]. Previous research by Ashida et al. [46] has shown that when individuals receive encouragement from other members of their network, it is associated with higher motivation to engage in health screenings. Furthermore, Ashida et al. [47] demonstrated that older adults are more likely to consume and stick with a healthy diet if their peers do [48]. A systematic review by Burton et al. [49], found that the most frequently identified motivators for older people to participate in training activities were social support and engagement with older peers. Peer-led exercise programs also were reported to be as effective as those led by a professional instructor [50].

In an aged care context, group activities and group settings are commonplace [10]. Older adults’ peers often share similar characteristics—for example, age, life experience and similarities in history (Figure 1b). Peers also have an enhanced capacity to share, relate and empathise with their group in a way that non-peers are often not able to [51]. According to Simoni et al. [52], an aged care facility needs to have a well-managed peer program, in which peers are valued and clinicians are trained to deliver specific interventions to residents together. In our context, meaningful seated activities like bingo, listening to music or drawing, then it is quite possible that older adults develop more positive attitudes towards using a social robot.

Meanwhile, previous research by Wu et al. [27], has highlighted the presence of fear amongst older adults of losing human contact because of the introduction of social robots in a care setting. This highlights the need to ensure that any intervention involving a social robot must be effectively planned and managed, and adequately addresses any concerns of older adults.

**Proposition** **3.** *Older adults are more likely to use a social robot if they are adequately introduced to and encouraged by important members of their social network in an aged care facility to use the technology.*

### 5.4. Facilitating Conditions

Facilitating conditions relate to whether an individual believes that the infrastructure within an organisation exists to use a system or technology [32]. Facilitating conditions such as the availability of help, support, training, and the like help older adults in adopting new technology.

Older adults in an aged care environment expect support while using new technology. Older adults have reportedly been concerned about operating devices and the implications around the malfunction of technology [27]. At home, informal sources of support may come from the immediate family, friends, and neighbours [53]. However, in a formal, aged care setting it would be expected that professional support is available in learning, continued use, maintenance, and repair of any technology-related devices [54].

It is well-recognised that resource constraints—at times—limit the deployment and effective use of technology in aged care. Organisations may face financial constraints in procuring and maintaining technology. Tight budgets may also limit the number of staff who are available to run technology-based activity programs. Moreover, there could be constraints on staff time which may result in a preference for group activities over single-user technology-based activities (such as interacting with a social robot). Finally, staff may also need time to not only facilitate activities but also learn how to use the technology [55].

**Proposition** **4.** *When older adults feel assured that there is adequate technical help and support available, they will be more likely to use a social robot for meaningful seated activities.*

### 5.5. Behavioural Intentions to Use Social Robots

The stronger an individual’s intention to engage in a behaviour, the more likely that the behaviour will be performed [56]. Therefore, if an older adult is strongly motivated to use a social robot for a particular task, chances are that he or she will do so.

On the other hand, the intention-behaviour gap is a reality [56]. Despite older adults’ intentions to use a robot, the behaviour may not be performed. As has been argued there could be a range of external factors—such as a power failure—which may inhibit an individual’s adoption of a new technology [56], even though the intention was there. However, given researchers’ agreement that intention is a significant antecedent of behaviour, it is proposed that:

**Proposition** **5.** *Older adults’ use of a social robot will be driven by their intention to use this technology.*

## 6. Limitations

This publication is entirely theoretical. As it seeks to explore and expand upon the emerging theoretical literature regarding the acceptance and use of social robots, older adults and meaningful seated activities, there are no research findings. This has limited this publication to an exploration of the existing literature and a series of theoretical propositions based on this phenomenon.

## 7. Conclusions

Research on social robots, older adults and meaningful seated activities in Australia is still emerging. To effectively explore and understand this phenomenon, it has been the contention of the researchers to apply the UTAUT model to these concepts. This study has sought to contribute to the emerging literature by exploring if each expected proposition for each concept of the UTAUT model could be satisfied when applied to older adults and social robot-initiated, meaningful seated activities in an Australian care setting. This publication is entirely theoretical and sets the foundations for future exploratory research regarding this phenomenon in a practical setting.

## Data Availability

Data are contained within the article.
